# Testing the regulatory framework in South Africa – a single-blind randomized pilot trial of commercial probiotic supplementation to standard therapy in women with bacterial vaginosis

**DOI:** 10.1186/s12879-020-05210-4

**Published:** 2020-07-10

**Authors:** Anna-Ursula Happel, Ravesh Singh, Nireshni Mitchev, Koleka Mlisana, Heather B. Jaspan, Shaun L. Barnabas, Jo-Ann S. Passmore

**Affiliations:** 1 Division of Medical Virology, Faculty of Health Sciences, Institute of Infectious Disease and Molecular Medicine, University of Cape Town, Anzio Road, Observatory, Cape Town, 7925 South Africa; 2 NRF-DST CAPRISA Centre of Excellence in HIV Prevention, Cape Town, South Africa; 3 School of Laboratory Medicine and Medical Sciences, University of Kwa-Zulu Natal, Durban, South Africa; 4 National Health Laboratory Service, Durban, South Africa; 5 Seattle Children’s Hospital, Seattle, USA; 6 Family Centre for Research with Ubuntu, Stellenbosch University, Tygerberg, South Africa

**Keywords:** BV, Probiotics, Regulation, South Africa, SAHPRA, Vaginal health, Randomized trial

## Abstract

**Background:**

Bacterial vaginosis (BV) increases HIV risk and adverse reproductive outcomes. Standard-of-care (SOC) for BV are antibiotics; however, cure rates are low. Probiotics for vaginal health may be useful in improving cure and recurrence although the regulatory framework governing probiotics and the conduct of randomized clinical trials to evaluate these has not been established in South Africa. We performed an exploratory single-blind trial evaluating a commercial oral-vaginal-combination probiotic as adjunct to SOC for BV treatment.

**Methods:**

Women with symptomatic vaginal discharge were screened for BV and common sexually transmitted infections (STIs). BV+ (Nugent 7–10) but STI- women were randomized to vaginal metronidazole alone (*n* = 12) or to metronidazole followed by a commercial oral/vaginal probiotic (*n* = 18). The primary qualitative outcome was to test the regulatory landscape for conducting randomized probiotic trials in South Africa; and acceptability of vaginal application by women. BV cure at 1 month (Nugent≤3) was the primary quantitative endpoint. Secondary quantitative endpoints were BV recurrence, symptoms, vaginal microbiota and genital cytokine changes over 5 months post-treatment.

**Results:**

The  South African Health Products Regulatory Authority (SAHPRA) reviewed and approved this trial. As probiotics continue to be regulated as health supplements in South Africa, SAHPRA required a notification application for this trial. Acceptability and adherence to the oral and vaginal application of the probiotic were high, although women reported a preference for oral capsules. 44.8% of women cleared BV one-month post-treatment, and no significant differences in BV cure (RR = 0.52, 95% CI = 0.24–1.16), recurrence, vaginal pH, symptoms, microbiota or vaginal IL-1α concentrations were found between SOC and intervention groups in this pilot study with an over-the-counter product.

**Conclusion:**

Navigation of the SAHPRA registration process for evaluating a commercial probiotic in a randomised trial laid the foundation for planned larger trials of improved probiotic products for vaginal health in South Africa. Although adherence to the vaginally delivered probiotic was high, women preferred oral application and we recommend that improvements in the content and method of application for future probiotics for vaginal health should be considered.

**Trial registration:**

This trial was registered on 17 October 2017 with the South African National Clinical Trial Register (http://www.sanctr.gov.za/; BV-trial1; DOH-27-1117-5579).

## Background

Bacterial vaginosis (BV) is the most common genital condition of women of reproductive age [1], defined by a rapid shift in the composition of vaginal bacterial communities from a *Lactobacillus*-dominated to a polymicrobial microbiota, compromising a mixture of diverse anaerobes, such as *Prevotella*, *Gardnerella*, BVAB1, *Sneathia*, and *Megasphera* spp. [2–4]. It increases risk of adverse pregnancy outcomes [5, 6] and acquisition and transmission of sexually transmitted infections (STIs), including HIV [7, 8], possibly due to the associated genital inflammation [9]. Antibiotics remain the standard-of-care (SOC) for treating BV although more than 50% of women experience recurrent episodes within 6–12 months [10, 11]. While most countries treat BV as part of syndromic management [12], more than 85% of BV cases are asymptomatic but nonetheless associated with significantly elevated genital inflammation [13]. In Africa, where epidemics of BV, STIs and HIV converge [14] and genital inflammation associated with even asymptomatic BV may increase HIV risk [8, 15], an urgent need to rethink and improve the SOC for treating BV exists.

Several clinical studies have evaluated *Lactobacillus*-containing probiotics as an adjunct to antibiotics in treating BV [16, 17]. However, there is clinical equipoise as to whether adjunctive probiotics improve BV cure and/or recurrence rates [18]. As the microbial composition of the lower female genital tract (FGT) in health and dysbiosis may be geo-adapted, with regional differences in diet, vaginal insertion, hygiene practices and host genetics possibly shaping these [19–21], it is critical to conduct trials in South Africa to test the effectiveness of probiotics for vaginal health in a local context. While randomized controlled trials (RCTs) assessing the effects of probiotics on BV management have been performed in several other African countries [22, 23], only one exploratory pilot study has recently been performed in South Africa, although with a European product that is not locally available [24].

In South Africa, the registration of medicines, probiotics and health supplements along with their use in clinical trials is regulated by the South African Health Products Regulatory Authority (SAHPRA), but none of the > 100 currently available over-the-counter (OTC) probiotics [25] has previously been formally reviewed by SAHPRA and no trial evaluating the impact of probiotics on health outcomes has been SAHPRA-approved or acknowledged. Gaining understanding of the regulatory framework for testing and introducing new vaginal geo-adapted probiotics intended for vaginal application is critical. Here, we describe the process and acceptability of conducting an exploratory single-blind trial in South African women diagnosed with BV. The main qualitative outcome was to test the South African regulatory framework and local acceptability of conducting probiotic trials for BV with vaginal application. BV cure in women receiving probiotic in adjunct to SOC compared to SOC only was an exploratory quantitative outcome, together with understanding of adherence, changes in vaginal pH, clinical symptoms, vaginal cytokine concentrations and bacterial microbiota in both groups.

## Methods

### Regulatory approval

This trial was submitted and approved by SAHPRA (SAHRPA Ref 20,161,201; PI: S. Barnabas), the University of Cape Town’s (UCT) Human Research Ethics Committee (HREC Ref 706/2016) and the South African National Health Research Ethics Council (NHREC Ref 4579) and registered with the South African National Clinical Trials Register of the Department of Health (DOH-27-1117-5579).

### Eligibility criteria

Women were recruited from a South African public sector STI clinic (Spencer Road Clinic) and from the UCT Student Wellness Centre in Cape Town, South Africa. Eligible women were 18–45 years old and seeking care for vaginal discharge. All eligible women were tested for BV (by Nugent scoring) and STIs, including *C. trachomatis, N. gonorrhoea, T. vaginalis,* and *M. genitalium* by TaqMan® Assays (Fast Track Diagnostics). Inclusion criteria were being BV positive (Nugent 7–10) but negative for any STI. Exclusion criteria included being pregnant, breastfeeding, pelvic inflammatory disease, living with HIV, having a known allergy to metronidazole, and/or currently using any other antibiotics or natural remedies in the urogenital area. Women who acquired an STI or had recurrent BV over the course of the trial were referred for treatment but not excluded, and any concomitant medication (including antibiotics) taken over the course of the study was recorded. Study visits were planned such that women were not menstruating nor reported having unprotected sex or douching in the 48 h prior to sampling. All women were tested for HIV (Rapid Anti-HIV (1&2) test; InTec products, Inc., China) and pregnancy (hCG Pregnancy test; Homemed™, South Africa) at screening.

### Sample size calculations

This was an exploratory study designed to establish the regulatory landscape to conduct probiotic trials in South Africa, with additional exploratory quantitative analyses. We based our sample size calculation of 30 women on cure rates from previous studies [22, 26]. Petricevic et al. (2008) reported a decline in BV in 83% of intervention compared to 35% in control arm participants after oral application of 300 mg clindamycin for 7 days followed by vaginal capsules containing 10^9^ CFU of *L.casei rhamnosus* for 7 days and Anukam et al. (2006) reported cure rates of 88% in the treatment group and 40% in the control group after administering 500 mg metronidazole orally for 7 days followed by oral administration of *L.rhamnosus* and *L.reuteri* (each 10^9^ CFU) for 30 days. In both studies, cure rates were assessed 30 days after the last administration of the probiotics. Since we applied probiotics both orally and vaginally and for 15 days, we expected the cure rates to be similar. Thus, we anticipated a sample size of 30 women (enrolled in a 2:3 ratio in SOC: intervention arms) would detect the above difference in proportions (0.83 vs. 0.35) with 75% power at α = 0.05, which we considered sufficient for this pilot trial.

### Randomisation and blinding

Randomisation was performed using the pseudorandom number generator in Microsoft Excel 2016 (MT19937) by research pharmacists at the UCT CRC who were not involved with clinical procedures and/or screening processes and only dispensed the investigational product. Researchers and laboratory staff involved in sample and data analysis were blinded to the randomisation process. The research nurse who conducted the clinic visits was not blinded as she interacted directly with participants. After the database lock and a primary blinded analysis, the unblinded treatment allocations were released.

### Dosing regimens

Eligible women either received topical metronidazole only (0.75% gel, 5 g vaginally, once a day for 5 days) or topical metronidazole followed by a 15-day treatment course of a probiotic marketed in South Africa to improve vaginal health (intervention group), which included 5 days of oral probiotic capsules followed by 10 days of oral capsules together with twice daily vaginal spray. The oral capsules and each metered dose of the vaginal spray contained lyophilized *L. acidophilus, L. rhamnosus GG, B. bifidum* and *B. longum* at ≥2 × 10^9^ colony-forming units (CFU).

### Laboratory quality control of the probiotic lot used in the study

The contents and concentrations of each microbial species of the probiotic lot were confirmed prior to initiation of the trial (Additional Figure 1). Briefly, one full oral or vaginal dose was dissolved in Brain Heart Infusion broth (supplemented with 0.1% starch and 1% yeast, sBHI), serially diluted and plated in triplicates onto sBHI agar plates. Plates were incubated at 37 °C for 48 h under anaerobic conditions (using Oxoid™ AnaeroGen™ 2.5 L Sachets, Thermo Fisher Scientific Inc., USA). The CFU per well were counted and the average concentration per dose was calculated. Contents were confirmed by MALDI-TOF (MALDI Biotyper, Bruker Daltonik, USA).

### Clinical procedures and sample collection

At screening, a vulvo-vaginal swab for STI testing and a posterior fornix and lateral wall swab to screen for BV by Nugent Scoring were collected. At enrolment (pre-treatment), and 1, 3 and 5 months post-treatment, genital samples were collected in the following order under speculum examination: (1) a vulvo-vaginal swab for STI testing; (2) a posterior fornix and lateral wall swab to screen for BV (by Nugent Scoring) and to measure vaginal pH (using a colour-fixed indictor pH strip; Macherey Nagel); and (3, 4) two lateral wall swabs to measure genital IL-1α (as marker of genital inflammation, by ELISA) and vaginal microbiota (by qPCR). In addition, women completed a questionnaire on demographics, reproductive health and sexual behaviour at enrolment, and a questionnaire assessing feasibility, acceptability and adherence to the administered products, vaginal symptoms and adverse events at follow up visits. Product preference was assessed at the final visit by questionnaire (Additional File 1). Adherence was measured by self-report in medication diaries and questionnaires, as well as return of empty packages at the one-month follow-up visit.

### Testing for STIs and BV

The commercial TaqMan® FTD STD9 (Fast Track Diagnostics, Luxembourg) kit, performed as per manufacturer’s instructions, was used to test for *N. gonorrhoeae*, *C. trachomatis*, *T. vaginalis* and *M. genitalium*. As positive controls, genomic DNA extracts from the following ATCC® strains were included: *N. gonorrhoeae* (ATCC® 700825), *C. trachomatis* (ATCC® VR-885), *T. vaginalis* (ATCC® 30001) and *M. genitalium* (ATCC® 33530). BV was diagnosed by Gram staining of vaginal smears and Nugent Scoring. Slides were assessed microscopically and assigned a score between 0 and 10, with a score of 0–3 considered BV negative, 4–6 intermediate microbiota, and 7–10 BV positive.

### Clinical outcomes

While this study was intended to test the regulatory landscape to conduct probiotic trials in South Africa and acceptability/preference of the probiotic administration for vaginal health, BV cure rates were considered (defined as having Nugent≤3 one-month post-treatment) as primary quantitative endpoint. In addition, other secondary endpoints included adherence, recurrence, changes in vaginal pH, concentrations of bacterial species contained in the administered probiotic (*L. acidophilus, L. rhamnosus, B. bifidum* and *B. longum*) and bacterial species associated with vaginal health (*L. crispatus, L. jensenii, L. iners, L. gasseri, L. vaginalis, L. mucosae*) or BV (*G. vaginalis, P. bivia*, BVAB2, *Megasphaera* 1 and *A. vaginae*), and IL-1α concentrations (as a bio-marker of genital inflammation) over 5 months post-treatment.

### Measuring vaginal bacterial concentrations

Concentrations of vaginal *Lactobacillus* spp. (including *L. crispatus, L. jensenii, L. iners, L. gasseri, L. vaginalis, L. mucosae*), BV-associated organisms (including *G. vaginalis, P. bivia*, BVAB2, *Megasphaera* 1 and *A. vaginae*), and bacterial species contained in the administered probiotic product (including *L. acidophilus, L. rhamnosus, B. bifidum* and *B. longum*) were assessed using commercially available Applied Biosystems™ TaqMan® Assays (Thermo Fisher Scientific Inc., USA; Assay IDs Ba04646245_s1, Ba04646258_s1, Ba04646257_s1,, Ba04646234_s1 for *Lactobacillus* spp. and Ba04646236_s1, Ba04646278_s1, Ba04646229_s1, Ba04646230_s1, Ba04646222_s1 for BV-associated species). For *L. vaginalis* (KF875988.1) and *L. mucoasae* (NR_024994.1) Thermo Fisher Scientific designed custom probes based on the referenced nucleotide sequence. The TaqMan probe sequences for *L. acidophilus* and *L. rhamnosus* (Haarman and Knol [27])*,* as well as *B. bifidum* and *B. longum* (Haarman and Knol [28]) were previously published. Using an ABI 7500 Real-Time PCR Detection System (Thermo Fisher Scientific Inc., USA), we quantified each of the targets. The quantification was performed using amplicons generated from plasmids for each of the targets (TaqMan™ Vaginal Microbiota Extraction Control). Serial dilutions, ranging from 10^0^ to 10^9^ molecules per μl of sample were used to generate standard curves. Bacterial concentrations were normalized to 16S rRNA gene concentrations, as recommended by the manufacturer.

### Measuring cervicovaginal IL-1α concentrations

Samples were thawed on ice and filtered by centrifuging at 1950 g for 10 min at 4 °C in SPIN-X® 0.2 μM cellulose acetate filters to exclude mucus and debris prior to performing the ELISA. Human IL-1α concentrations were measured using a commercial ELISA (E-EL-H008, Elabscience®, USA), according to the manufacturer’s instructions. IL-1α concentrations were calculated based on the standard curve, using standards provided with the kit. The detection range was 1.25–125 pg/mL, and all values below the detection limit were recorded as half of the lowest concentration measured.

### Statistical analyses

GraphPad Prism6® (GraphPad Software, USA), STATA version 11.0 (StataCorp, USA) and R were used for descriptive and statistical analyses and to generate graphs. Planned description of continuous variables with means, medians and standard deviations, as appropriate, were calculated. Categorical variables were described as proportions. Mann-Whitney U tests were used to compare groups of continuous variables and relative risk (RR) was used for categorical variables. 95% confidence intervals and *p*-values ≤0.05 were used to determine statistical significance.

## Results

### Path to SAHPRA approval

This trial intended to explore the regulatory environment in South Africa governing probiotic trials, in order to lay the foundation for future trials of novel probiotic products. Prior to conducting this randomized trial, no probiotic trial had been approved by the regulatory authorities in South Africa. Thus, discussions with SAHPRA authorities were initiated in March 2016, as a decision on whether a full application for the trial was necessary, given that this was a randomized trial with a vaginally applied product, or whether a notification application was required. Currently, already available OTC probiotics (even those applied topically) are not considered or regulated as a medicine in South Africa but rather as a health supplement. Typically, the regulator requires full applications for products not registered in South Africa or for those not being used for their registered indication, dose, or formulation. Alternatively, SAHPRA notification is required for phase IV clinical studies of an approved medication within its approved dosage, formulation and indication. SAHPRA finally confirmed that the trial did not require a full application but rather a notification application, which was submitted to SAHPRA and approved (SAHRPA Ref 20,161,201).

### Cohort behavioural and biomedical characteristics

**Fig. 1:**
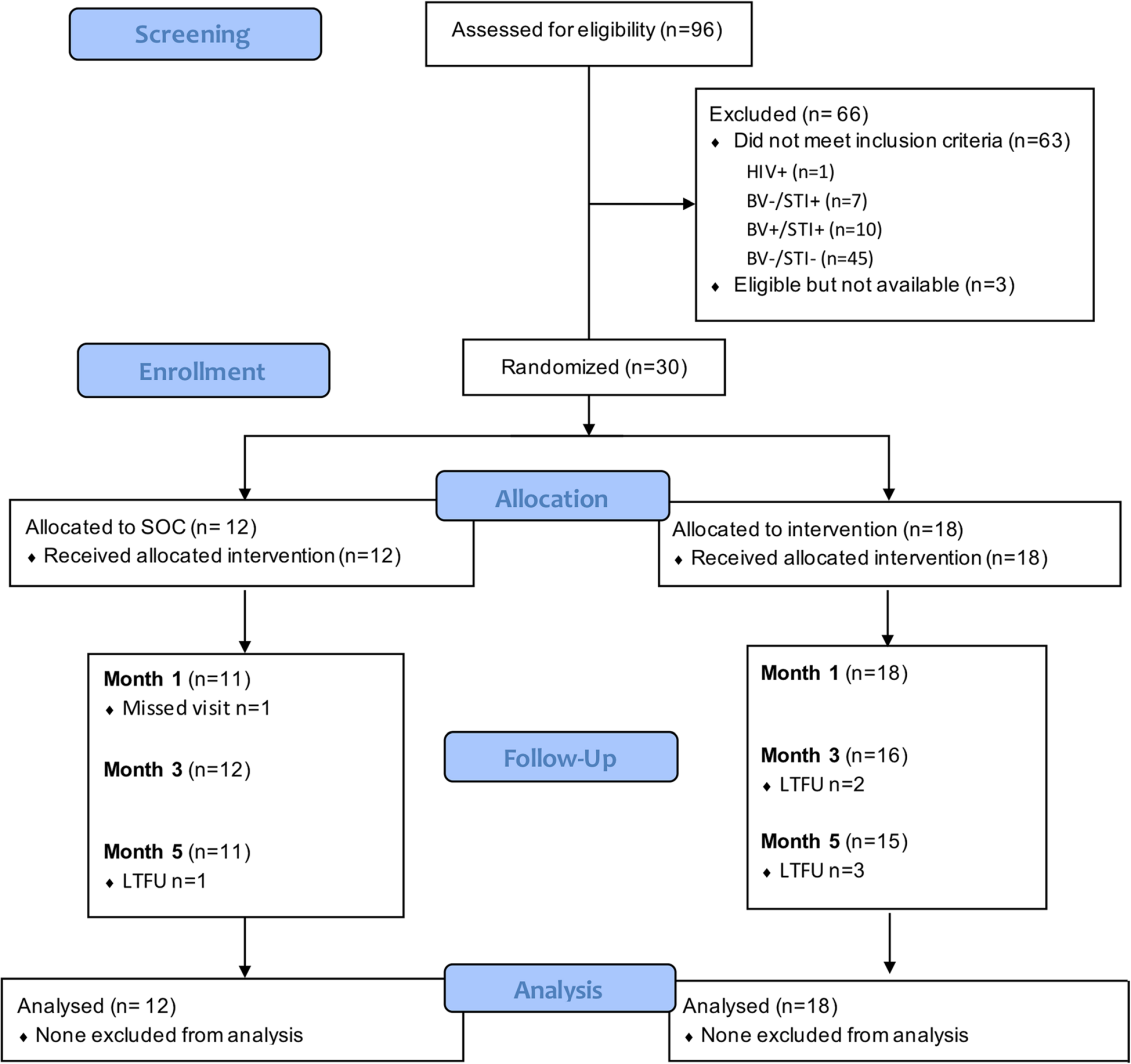
Consort diagram. A total of 96 women with symptomatic discharge were screened for eligibility Participants were tested for BV by Nugent Scoring on for STIs (including *C. trachomatis, T. vaginalis, M. genitalium* and *N. gonorrhoeae*) by Multiplex PCR. Eligible participants (BV positive but STI negative) were randomly assigned to the SOC (metronidazole only) or intervention arm (metronidazole plus probiotic). Follow-up visits took place 1, 3 and 5 months post-treatment. LTFU = lost to follow up

Between 30 October 2017 and 22 March 2018, a total of 96 women seeking care for vaginal discharge were screened for eligibility (Fig. 1), of which the majority (*n* = 90) were recruited via the UCT Student Wellness Centre. One woman tested HIV positive at screening, and thus was excluded and referred for care. Of 95 women who were eligible for screening, 43 women (45.3%) were confirmed to have BV (Nugent 7–10; Table 1). Given that we recruited symptomatic women, this ratio confirms that vaginal discharge is a very imprecise tool for predicting the presence of BV [14], with a positive predictive value of only 45.3% in this cohort. In addition, 17.9% (17/95) had an STI, with *T. vaginalis* (9.5%, 9/95) and *C. trachomatis* (8.4%, 8/95) being the most common STIs detected. Of the 43 BV positive women, 33 were eligible for randomization (BV+ but STI-).

**Table 1:** Screening results

Age participant [years, median (IQR)]	22 (20–25)
**Self-identified race [% (n/N)]**	
Black	67.7 (65/96)
Coloured	19.8 (19/96)
White	12.5 (12/96)
**Bacterial vaginosis** (Nugent Score 7–10) **[% (n/N)]**^**a**^	45.3 (43/95)
**Bacterial and protozoal infections [% (n/N)]**^**a**^	
*T. vaginalis*	9.5 (9/95)
*C. trachomatis*	8.4 (8/95)
*M. genitalium*	3.2 (3/95)
*N. gonorrhoea*	2.1 (2/95)
**Eligible (BV+/STI-) [% (n/N)]**^**a**^	34.7 (33/95)

^a^One participant tested HIV positive, thus screening was aborted

Of the 33 women who were eligible, three were not interested in participating further. Therefore, 12/30 were randomized to the SOC arm (vaginal metronidazole alone; 5 days), while 18/30 were randomized to the intervention arm (vaginal metronidazole for 5 days, followed by 15 days of oral/vaginal probiotics). Randomised women were a median age of 22 years (IQR 20–26 years) old, predominantly single (26/30, 86.7%), and self-identified as black (20/30, 66.7%). Their median age at sexual debut was reported to be 17 years (IQR 16–19 years) with a median of five lifetime sexual partners (IQR 3–8), and reporting of oral sex was common (21/30, 70.0%). About half (18/30) reported regular condom use, and few (*n* = 4) reported previously being diagnosed or treated for an STI (Table 2). Ninety-three percent (28/30) reported a history of chronic vaginal discharge, and more than half (20/30) reported previous use of prescription medicine to reduce vaginal discharge and malodour. Almost half (14/30) reported that they were currently smoking, a factor that has previously been associated with the risk of BV [29–31]. Similarly, vaginal cleansing practices have been described as risk factor for BV [32–34], and more than half (17/30) reported practicing some kind of cleansing, including douching, using their fingers, water or soap to clean their vaginas internally (Table 2). More than half used tampons while menstruating, suggesting that they may likely be comfortable with the vaginal application of the probiotic (Table 2). None of the socio-behavioural characteristics evaluated differed by study arm.

**Table 2:** Cohort characteristics at enrolment

	SOC ***n*** = 12	Intervention ***n*** = 18
**Age participant [years, median (IQR)]**	23 (21–34)	22 (20–26)
**Self-identified race [% (n)]**		
Black	58.3 (7)	72.2 (13)
Coloured	25.0 (3)	16.7 (3)
White	16.7 (2)	11.1 (2)
**Age menarche [years, median (IQR)]**	12 (11–15)	14 (13–16)
**Marital Status [% (n)]**		
Single	83.3 (10)	88.9 (16)
Married	8.3 (1)	–
Separated/Divorced	8.3 (1)	11.1 (2)
**Pregnancies and contraception [% (n)]**		
Ever been pregnant	25.0 (3)	27.8 (5)
Currently using hormonal contraception	33.3 (4)	38.9 (7)
**Reported current smoking [% (n)]**	50.0 (6)	44.4 (8)
**Sexual risk behaviour and sexual health**		
Age of sexual debut [median (IQR)]	18 (17–18)	17 (16–19)
Number of lifetime partners [median (IQR)]	5 (3–8)	4 (3–8)
Oral sex during last 6 months [% (n)]	58.3 (7)	77.8 (14)
Regular condom use during last 6 months [% (n)]	66.7 (8)	55.6 (10)
Ever been diagnosed or treated for a STI [% (n)]	8.3 (1)	16.7 (3)
Partner or participant STI in last 6 month [% (n)]	–	16.7 (3)
Long-term history of vaginal discharge [% (n)]	83.3 (10)	100 (18)
**Vaginal product use [% (n)]**		
Use of medicine from doctor/nurse	58.3 (7)	72.2 (13)
Use of tampon	25.0 (3)	16.7 (3)
Cleansing with fingers	16.7 (2)	11.1 (2)
Cleansing with water	25.0 (3)	33.3 (6)
Douching to clean vagina	–	16.7 (3)
Cleansing with soap	8.3 (1)	5.6 (1)

### Adherence and safety

Women attended a total of 143 visits, with the majority (25/30) completing all visits (Fig. 1) and all women (30/30) completing the entire five-day course of metronidazole. All women randomised to the probiotic (18/18) completed the course of oral probiotic capsules, and the majority (16/18) also completed the vaginal probiotic spray.

Overall, a total of 110 adverse events (AEs) were recorded over the course of the study. Most were mild (WHO grade 1–2, 109/110, 99.1%) and unrelated to study product use (76.4%; 84/110), including headaches (*n* = 20), flu-like symptoms (*n* = 18), STI acquisition, including *C. trachomatis* (*n* = 8), *T. vaginalis* (*n* = 7), *M. genitalium* (*n* = 5) and *N. gonorrhoea* (*n* = 1), menstrual pain (*n* = 7), gastro-intestinal complaints (*n* = 5), temporary vaginal discomfort related to vaginal intercourse (*n* = 7), anaemia (*n* = 2), cat allergy (*n* = 1), buttock pain (*n* = 1), and anxiety (*n* = 1). It was concerning that more than half of the women (53.3%, 16/30) acquired an STI over the course of the study, supporting that BV is considered a risk factor for acquisition of STIs [7, 8]. Although BV has also been described as risk factor for urinary tract infections [35], none were reported by the participants throughout the course of the study.

Of AEs considered to be related to study products (*n* = 26), the majority was associated with vaginal metronidazole use and included previously describe side effects of vaginal metronidazole gel [36], including vaginal itching/irritation (9/30, 30.0%), candida infections (5/30, 16.7%), discomfort (3/30, 10.0%), increased discharge (2/30, 6.7%), spotting (1/30, 3.3%), or constipation (1/30, 3.3%). One third of the women administering probiotics (5/18, 27.8%) reported AEs after vaginal probiotic use, including vaginal itching/discomfort (2/18, 11.1%), increased vaginal discharge (1/18, 5.6%), increased nipple sensitivity (1/18, 5.6%), and nausea (1/18, 5.6%).

### Acceptability

**Fig. 2:**
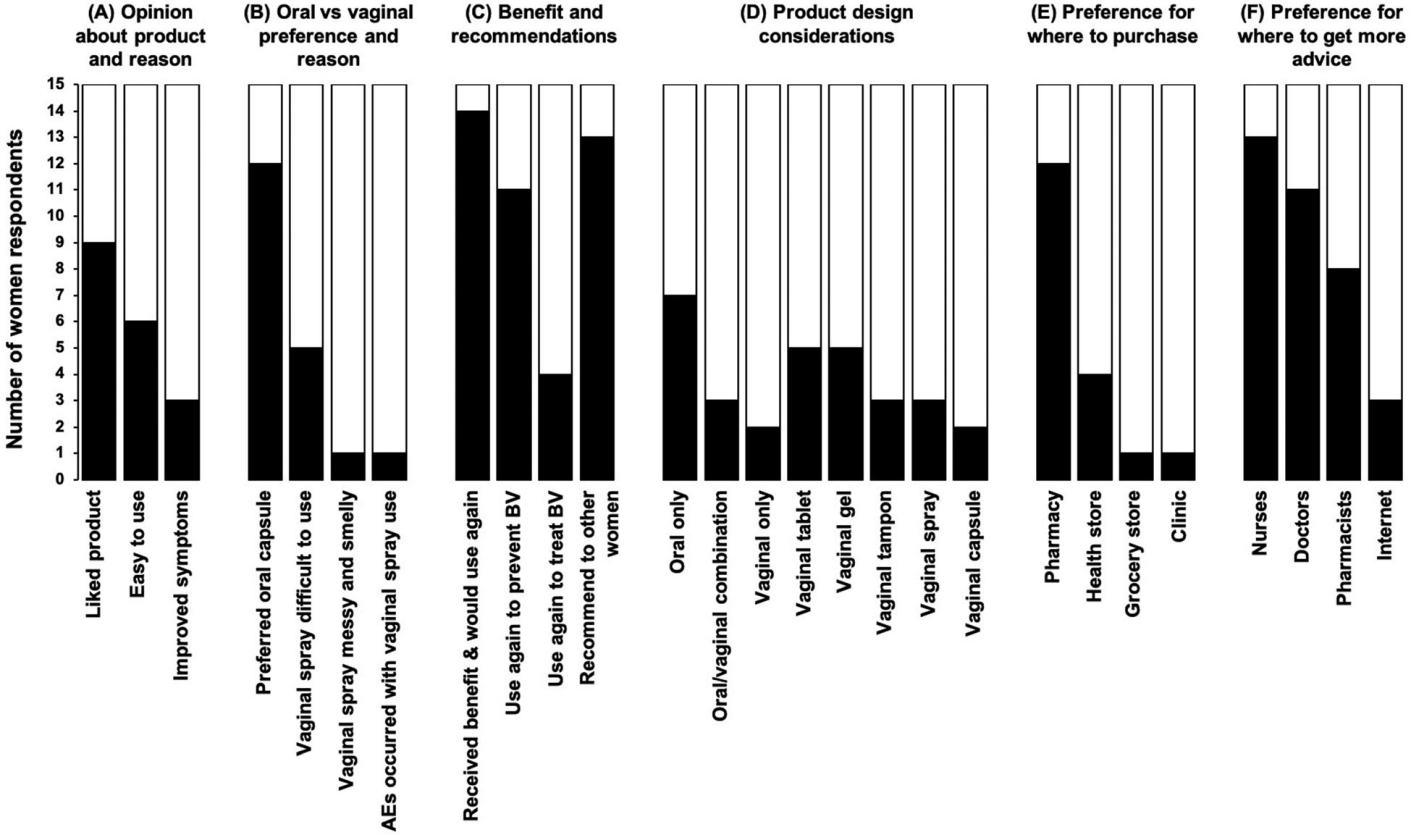
Acceptability and preference of probiotics for vaginal health. At the final visit, participants completed a questionnaire to assess their (**a**) opinion about the oral/vaginal probiotic they used during the trial, (**b**) why their preferred the oral or vaginal application, (**c**) their perceived benefits and whether they would use it again or recommend it to another woman, (**d**) their application preference for the development of a future probiotic for vaginal health, (**e**) where they would want to buy it and (**f**) from whom they would like to get advice regarding the use of probiotics for vaginal health

More than half of the women (9/15, 60.0%) reported that they liked using the combination probiotic because it was easy to use or resulted in improved vaginal symptoms (Fig. 2a). Most women (12/15, 80.0%) reported that they preferred the oral capsule over the vaginal spray (Fig. 2b). Women reported that the vaginal spray was difficult to use, “messy and smelly” or that AEs occurred. The vast majority (14/15, 93.3%) believed that they received some benefit from this product and would buy it, primarily to prevent rather than to treat BV (Fig. 2c). The majority (13/15, 86.7%) also said they would recommend it to other women. In terms of future product design, women reported that they would prefer a probiotic with oral application rather than a combination of oral and vaginal or vaginal only administration (Fig. 2d). If vaginal administration was required, women responded that they would prefer a tablet or gel rather than a tampon, spray or capsule. Women reported that they would prefer to buy the probiotic at pharmacies (12/15, 80.0%) compared to health stores, a grocery store or clinic (Fig. 2e). Women reported a preference for asking nurses (13/15, 86.6%) or doctors (11/15, 73.3%) for advice about using probiotics for vaginal health rather than pharmacists or self-help on the internet (Fig. 2f). It was also important to note that none of the women reported to be willing to spend more than 200 South African rand (ZAR; ~USD11.40; 1:0.06), which was less than the cost of the probiotic tested in this trial (costing ZAR280; ~USD16.00).

### Comparing clinical outcomes in SOC and intervention arms

This trial was intended as an exploratory study to test the local regulatory environment and acceptability of vaginal probiotics. However, we also conducted an exploratory intention-to-treat (ITT) analysis to estimate possible benefits of administration of this locally sourced probiotic on clinical outcomes. The majority of women reported an improvement of their vaginal symptoms post-treatment, including discharge, colour and odour, with similar rates in the SOC and intervention arms (Table 3). Of these, the majority reported a decrease of vaginal discharge, and more than half reported a change in smell and/or colour of the discharge, independently of treatment arm.

**Table 3:** Reported vaginal symptoms post-treatment

	Month 1			Month 3		
	SOC	Intervention	RR (95%CI)^**a**^ ***p***-value	SOC	Intervention	RR (95%CI)^**a**^ ***p***-value
**Discharge improvement [%(n/N)]**	83.3 (10/11)	77.8 (14/18)	0.86 (0.63–1.17) *p* = 0.324	83.3 (10/12)	81.3 (13/16)	0.96 (0.69–1.34) *p* = 0.886
**Less discharge**	90.0 (9/10)	78.6 (11/14)	0.87 (0.62–1.23) *p* = 0.438	90.0 (9/10)	61.5 (8/13)	0.68 (0.42–1.10) *p* = 0.118
**Change in colour**	50.0 (5/10)	57.1 (8/14)	1.14 (0.53–2.46) *p* = 0.733	70.0 (7/10)	46.2 (6/13)	0.66 (0.32–1.35) *p* = 0.253
**Change in smell**	70.0 (7/10)	71.4 (10/14)	1.02 (0.60–1.72) *p* = 0.940	80.0 (8/10)	61.5 (8/13)	0.77 (0.45–1.31) *p* = 0.332

^a^Relative Risk (RR) and 95% Confidence interval (CI) were used to compare groups

**Fig. 3:**
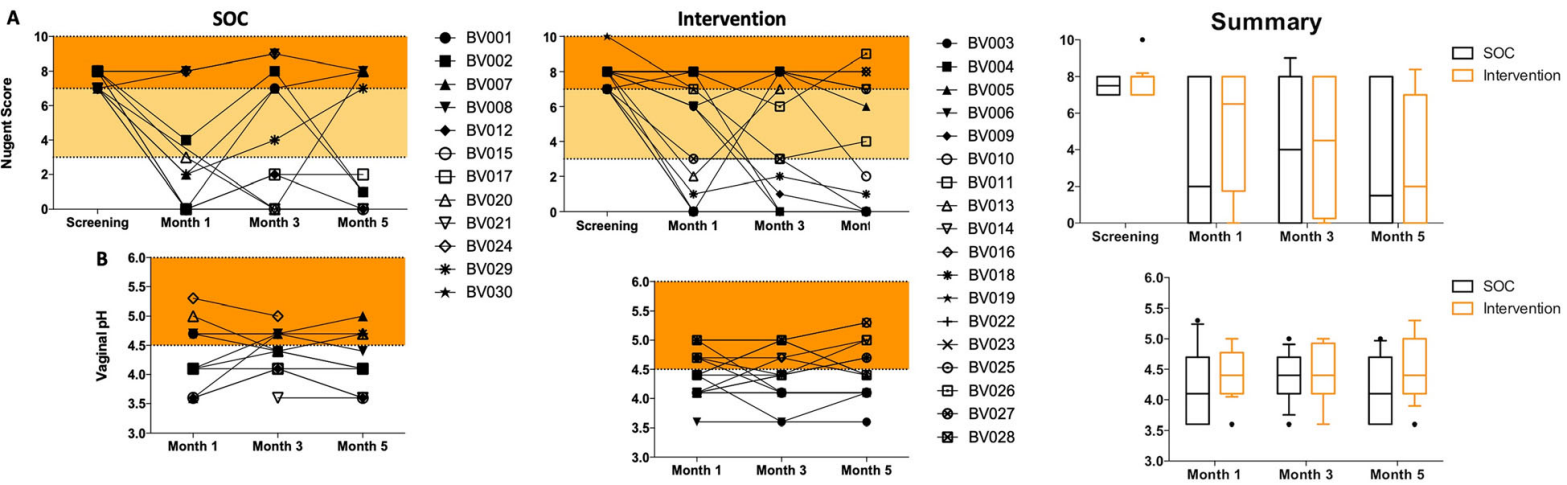
BV status and vaginal pH in the SOC and intervention group. **a** BV status was determined by Nugent scoring at screening, and one, three and five months post-treatment. Nugent Score 0–3 = BV negative (white area); 4–6 = BV intermediate (yellow) and 7–10 = BV positive (orange). **b** Vaginal pH was measured using color-fixed indicator strips one, three and five months post-treatment. A vaginal pH < 4.5 (white area) is seen as protective. Each participant is represented by a symbol-coded dot, and the summaries show median and interquartile range

The overall BV cure rate (defined as achieving a Nugent score 0–3) was 44.8% at month one, 46.4% at month three and 53.8% at month five. BV cure rates tended to be higher in the SOC arm (63.6%; 7/11) compared to the intervention arm (33.3%; 6/18; RR = 0.52, 95% CI = 0.24–1.16, *p* = 0.109) 1 month post-treatment (Fig. 3a). However, almost half of the cured women in the SOC arm (3/7) subsequently re-tested BV positive (Nugent 7–10) at month 3, while more than half of the cured women in the intervention arm (4/6) remained BV negative until the end of the trial.

In the SOC arm, 6/11 (54.5%) women had a vaginal pH < 4.5 1 month after treatment, compared to 11/18 (61.1%) in the intervention group (RR = 1.12, 95% CI =0.58–2.15, *p* = 0.733; Fig. 3b). Similarly, no significant difference between the SOC and intervention arms were noted at later time points. Nugent scores correlated significantly with vaginal pH (Spearman rho = 0.71; *p* = 0.0001).

### Evaluating the presence of probiotic species in the genital tract

**Fig. 4:**
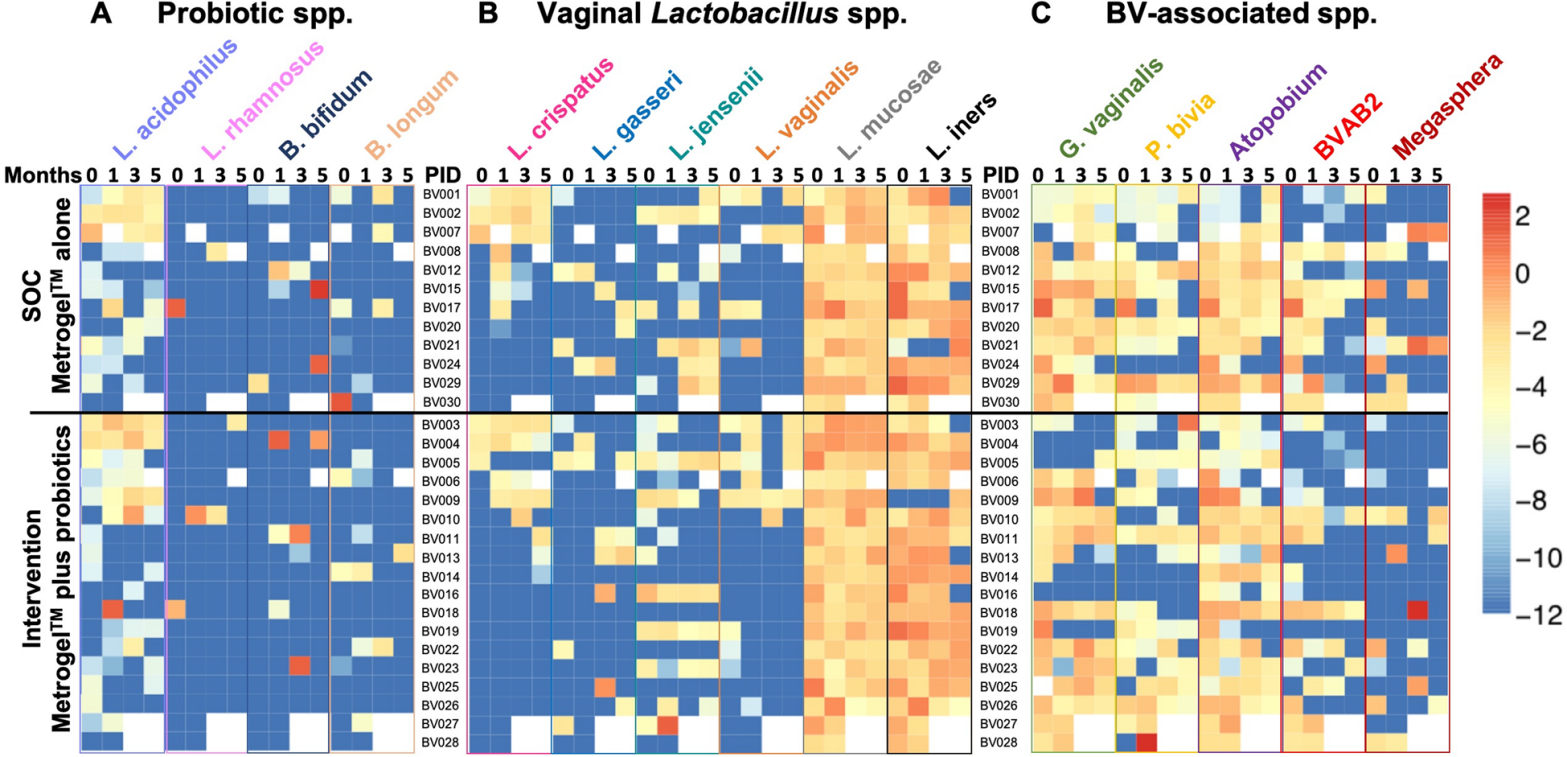
Quantities of vaginal bacterial species in the SOC and intervention group. **a** Bacterial species contained in the administered probiotic, including *L. acidophilus, L. rhamnosus, B. bifidum and B. longum,* vaginal *Lactobacillus spp.,* including *L. crispatus, L. gasseri, L. jensenii, L. vaginalis, L. mucosae* and *L. iners* (**b**) and BV-associated bacteria, *including G. vaginalis, P bivia, Atopobium,* BVAB2 and *Megasphera* (**c**) were measured by qPCR and normalised to total 16S rRNA gene concentration at month 0 (enrolment) and 1, 3 and 5 months post-treatment. Values were log-transformed and supervised clustering was used to generate the heatmap. Each row shows one participant. Darkest blue indicates levels below detection limit. White data points indicate missing data. Light purple indicates participants from the SOC and dark purple participants from the intervention group

To establish a framework on whether the bacterial species contained in the oral/vaginal probiotic colonised the FGTs of women, concentrations of *L. rhamnosus, L. acidophilus, B. bifidum* and *B. longum* were measured before and after treatment (Fig. 4a). The majority of women (20/30) had detectable concentrations of *L. acidophilus* at baseline, although few had detectable levels of *L. rhamnosus* (2/30)*, B. bifidum* (2/30) and *B. longum* (8/30). However, we found no evidence of colonization of any of the bacterial species contained in the probiotic formulation post-treatment (Fig. 4a).

### Commensal *Lactobacillus* and BV-associated communities following BV treatment

Most women had very low concentrations of *L. crispatus*, *L. gasseri, L. jensenii* and *L. vaginalis* before treatment, as expected (Fig. 4b). While some women showed increased concentrations of beneficial vaginal *Lactobacillus* spp*.* 1 month after treatment, others did not, and this did not differ by study arm. *L. mucosae* and *L. iners* were more commonly detected and concentrations were generally higher than those of other *Lactobacillus* spp. (Fig. 4b).

In line with their BV diagnosis, most women had detectable concentrations of *G. vaginalis* (26/30)*, P. bivia* (21/30) and *A. vaginae* (29/30) pre-treatment (Fig. 4c). The concentrations of these BV-associated bacteria remained high throughout the trial despite BV treatment, both in the SOC and intervention arm. While most women had BVAB2 (22/30) and some *Megasphera* (14/30) present pre-treatment, only some women lost or had decreased quantities of these bacterial species post-treatment, irrespective of study arm (Fig. 4c).

### Genital inflammation and interactions between the host and vaginal microbiota

**Fig. 5:**
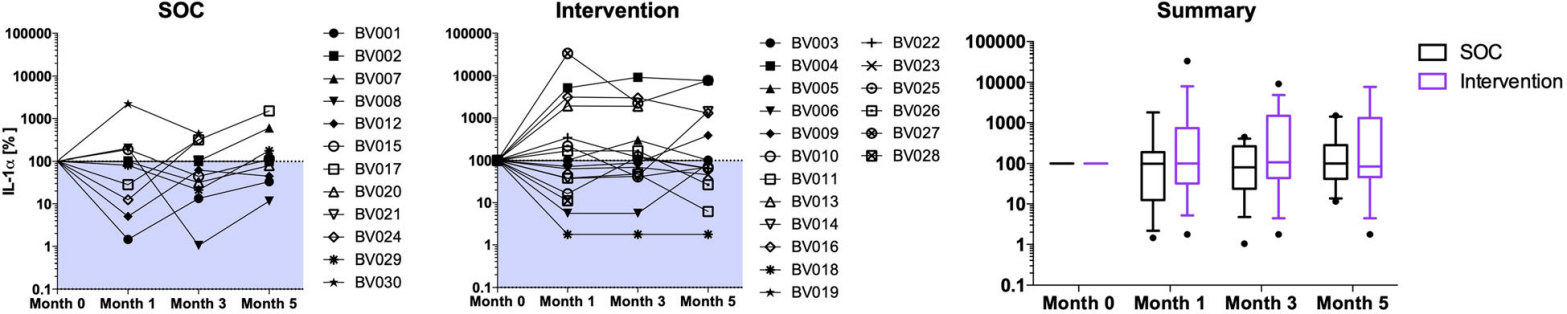
Levels of the inflammatory marker IL-1⍺ in the FGT of participants in the SOC and intervention arm. IL-1⍺ was measured by ELISA in FGT secretions of SOC and intervention group participants pre-treatment and 1, 3 and 5 months post-treatment. Each participant is represented by a symbol-coded dot. The percentages compared to the IL-1⍺ level measured pre-treatment (month 0) are displayed. The shaded area indicates a decrease in IL-1⍺ post-treatment. The summary shows the median and interquartile range

Genital IL-1α, a marker of inflammation [37], decreased after treatment in most women, albeit not significantly. However, levels of IL-1α increased again quickly to those measure pre-treatment (Fig. 5). Adjusting for STIs acquired during the trial had no impact on this observation. While IL-1α concentrations did not correlate with presence or absence of most *Lactobacillus* spp. and none of the BV-associated organisms, it correlated with *L. iners* concentrations 1 month (Spearman r = 0.368, *p* = 0.049), and 3 months post-treatment (Spearman r = 0.373, *p* = 0.055).

## Discussion

In the present study, we evaluated the regulatory environment for conducting probiotic trials in South Africa. SAHPRA required a notification application, as the probiotic was available OTC in South Africa and considered a health supplement. The use of a vaginal/oral probiotic combination was well accepted among South African women included in this exploratory study and was associated with few product-related AEs. While this study was not sufficiently powered to test efficacy of this convenient OTC probiotic in South African women, we observed no beneficial clinical effect of this adjunctive probiotic compared to vaginal metronidazole alone as SOC. Nonetheless, this exploratory trial provided valuable lessons for future probiotic trials of the same disease entity in the same population, with improved products and adequate sample sizes.

With regards to the regulatory landscape, SAHPRA regulates OTC probiotics currently as health supplements, independently of their route of administration and health claims being made, but labels must state that “This unregistered medicine has not been evaluated by the SAHPRA for its quality, safety or intended use” [38]. In contrast, all health claims for probiotics in the European Union have to be authorized by the European Food Safety Authority (EFSA). Any statement suggesting a relationship between a product and health outcomes is considered a health claim, including using the term “probiotic” on a product label [39]. Thus far, EFSA has rejected all submitted health claims for probiotics [39]. Further, vaginally applied probiotics used to be regulated as medical devices, with even stricter regulations, standards and certification processes than orally applied products. Recent changes in the Medical Device Regulation state that living organisms are no longer acceptable as ingredients for medical devices from 2020, thus currently marketed vaginally applied probiotic products will have to either be re-classified or transferred to a new product category [40]. In the United States, most probiotics are classified as foods or dietary supplements, which are required to comply with Good Manufacturing Practice guidelines, but no quality or efficacy testing is required [39]. As in the European Union, no disease-specific claims can be made on labels of dietary supplements, but functional claims, such as “improves vaginal health”, can be made when accompanied by a disclaimer [39]. Thus, while SAHPRA, the regulatory body in South Africa, seems to be more concerned with product safety than misleading claims, sound scientific approaches should be required to demonstrate specific health benefits of commercially available probiotics to more closely align with regulatory bodies in Europe and the United States. Previous South African studies have shown poor correlation between label claims and actual probiotics content [41], thus post-market surveillance should be mandatory as well.

With regards to product design, women reportedly preferred the oral over the vaginal application of the probiotic, as the administration of the vaginal spray was uncomfortable and messy. Thus, for future probiotic product design women’s preferences should be taken into consideration and probiotics containing well-selected vaginal *Lactobacillus* spp. should be administered orally. If topical administration is required, a vaginal tablet or gel would be preferred over a spray. Importantly, women were not willing to spend more than ZAR 200 (~USD11.40) for one treatment course. Given that > 60% of South African households earned less than ZAR 6367 (~USD368.55) per month according to the latest Census [42], the cost of one treatment course of the administered product per individual (ZAR 280; ~USD16.00) would use up 4.4% of the monthly income of those South African households. This highlights the need for the development of cheaper probiotics for vaginal health, making them available for those most in need of it.

The selected OTC oral/vaginal probiotic, like all those available in South Africa, does not contain *Lactobacillus* spp. that are commonly present in the FGT and associated with vaginal health, internationally and in South African women, such as *L. crispatus, L. gasseri* or *L. jensenii* [19, 43]. Others have shown that vaginal *Lactobacillus* spp. are highly adapted to this ecological niche [44], and since the product did not contain those highly adapted *Lactobacillus* spp., they might have failed to colonize, which is in agreement with our microbiological results. For a successful colonization and subsequent ability to confer a health benefit to the host, the bacteria need to fulfil a broad range of other criteria including adherence to vaginal epithelial cells, production of lactic acid, decrease of vaginal pH, decrease of inflammatory cytokines, and inhibition of BV-associated bacteria, such as *G. vaginalis* or *P. bivia* [43]. We have isolated and characterised the *Lactobacillus* strains contained in this OTC phenotypically and compared their characteristics to vaginal *Lactobacillus* strains from healthy South African women [43]. We did in fact find that vaginal *Lactobacillus* strains largely performed better in these in vitro assays than the strains currently used in probiotics for vaginal health, suggesting that it might not be ideal to include these strains in commercial probiotics for vaginal health.

Based on this experience with the South African regulatory body SAHPRA, we have some important insights for the design of future probiotic trials in South Africa. Clinical studies should test for BV cure shortly after treatment completion to minimize the likelihood that women have a recurrent BV event, in order to differentiate BV cure rates from confounding repeat episodes. Further, including a comparison with vaginal metronidazole gel alone or in combination with probiotic is more similar to a superiority trial rather than placebo-controlled [45], which is likely to make an efficacy trial with probiotics more difficult to power. Finally, we used vaginal metronidazole gel instead of oral metronidazole, which is more commonly used in South Africa for treating BV due to its lower cost. However, vaginal metronidazole gel is thought to be more effective and have less systemic side effects than the cheaper oral formulation [46, 47], which again should be taken into account when performing power calculations.

One interesting microbiological observation made during this study was that concentrations of vaginal *L. iners* correlated positively with the inflammatory cytokine IL-1α. This supports literature suggesting that *L. iners* is not necessarily as beneficial as other common vaginal *Lactobacillus* spp. [48–50]. *L. iners* is often present during BV, has complex nutritional requirements compared to other vaginal *Lactobacillus* spp., and encodes inerolysin, a pore-forming toxin that is related to *G. vaginalis*-encoded vaginolysin [50]. The high inflammation associated with *L. iners* in our study suggests that rather than only treating women with BV, also women with an *L. iners*-dominated microbiota and high genital inflammation could benefit from well-designed probiotics for vaginal health.

An important limitation of this study was the small number of participants; although this was intended as an exploratory study to test the regulatory environment in South Africa and the acceptability/preference of oral versus topical application. In addition, administration of the study drugs was self-reported and vaginal pH was not being measured pre-treatment. It was also unexpected that genital tract IL-1α levels differed at baseline between randomized groups, which is difficult to explain. Further, no 16S rRNA gene sequencing to evaluate relative vaginal bacterial abundance was performed, neither was bacterial culture.

## Conclusions

In a region with some of the highest rates of BV, STIs and HIV, it is critical that the local regulatory environment for conducting randomized trials of planned novel probiotic products is established. This randomized single-blinded trial provided important regulatory and clinical considerations critical in the design of future clinical trials, with larger sample sizes intended for safety and efficacy endpoints. This exploratory trial did suggest that oral and vaginal OTC probiotics were generally safe and acceptable in South African women with BV. Due to the critical need to manage BV better internationally and in South Africa, future double-blind, randomized, placebo-controlled trials, using a probiotic product containing beneficial, well-characterised vaginal *Lactobacillus* strains are needed to conclusively determine the efficacy of adjunctive probiotics on BV cure and recurrence in South African women.

## Supplementary information

**Additional file 1.** Participant questionnaires. Questionnaires administered to the participants to capture data on demographics, women’s reproductive and sexual health, use and preference of study products.

**Additional file 2: Figure S1.** Bacterial concentration per dose unit of the commercial probiotic. The CFU per dose of three boxes (pink, purple and red) of the probiotic lot containing oral capsules (C) and vaginal spray (S) was determined using serial dilutions and compared to the manufacturers claim of 2 × 109 CFU per dose (dotted line).

## Data Availability

The datasets used and/or analysed during the current study are available from the corresponding author on reasonable request.

## Abbreviations

BV: Bacterial vaginosis
BVAB2: Bacterial Vaginosis-Associated Bacterium 2
CFU: Colony-forming units
CRC: Clinical Research Centre
EFSA: European Food Safety Authority
FGT: Female genital tract
ICH: International Conference on Harmonisation
OTC: Over-the-counter
RCT: Randomized controlled trial
SA-GCP: South African Good Clinical Practice
SAHPRA: South African Health Products Regulatory Authority
sBHI: Brain Heart Infusion supplemented with 0.1% starch and 1% yeast
SOC: Standard of care
STI: Sexually transmitted infections
UCT: University of Cape Town
ZAR: South African Rand
