# Supplementary material for: Testing the regulatory framework in South Africa – a single-blind randomized pilot trial of commercial probiotic supplementation to standard therapy in women with bacterial vaginosis
Source: BMC Infect Dis. 2020 Jul 10;20:491. doi: 10.1186/s12879-020-05210-4 (PMC7350581; doi:10.1186/s12879-020-05210-4)
Supplement: Supplementary file 1 — Additional file 1. Participant questionnaires. Questionnaires administered to the participants to capture data on demographics, women’s reproductive and sexual health, use and preference of study products. [file 12879_2020_5210_MOESM1_ESM.pdf]

# Live biotherapeutics for treatment of bacterial vaginosis in HIV prevention in South African women – BV-trial1

**Principal Investigator:**
University of Cape Town, South Africa: Dr Shaun Barnabas

**Co-Investigators:**
University of Cape Town, South Africa: Dr Jo-Ann Passmore, Anna-Ursula Happel and Dr Heather Jaspan

**Funder:** Medicines Research Council

**University of Cape Town:**
Phone 021 650 7963

**Introduction**

Hello my name is ____________________. I am part of a research team from the University of Cape Town (UCT). In this study we would like to compare two different ways to treat bacterial vaginosis. When you have bacterial vaginosis, you have abnormal bacteria in your vagina. We also want to know about various factors in your life that may change your risk of getting infected with these abnormal bacteria. So, we are going to ask you questions about your sexual behaviour, substance use, education, social history and vaginal practices. This information will be kept confidential and your personal information will not be shared with anyone.

You have been invited as a possible participant because you are between 18 - 45 years old and have bacterial vaginosis. If you agree to participate in this study, please fill in the attached questionnaire. You will be asked to sign a consent form before you fill in this questionnaire, which you will get a copy of to keep. Please read the information on the consent form, and ask questions about anything you do not understand, before deciding whether you would like to participate or not.

# INTERVIEW DETAILS

**Participant Identification _____________**

**Date of Interview (DD/MMM/YYYY) _____________**

**Location of interview: _______________________**

**Interviewer Name: __________________________**

**Interview start time: (HH:MM AM/PM): ______________________**

**Interview stop time: (HH:MM AM/PM): ______________________**

# SECTION 1: DEMOGRAPHICS AND SOCIO-ECONOMIC STATUS

## *1.1: Demographics*

**What is your date of birth?** _______/________/______DAY/MONTH/YEAR

**What is your current marital status?**
*Select one.*
- ❑ Married (legal or traditional) or living as married
- ❑ Single
- ❑ Separated / Divorced
- ❑ Widowed
- ❑ Other, please specify: __________
- ❑ Prefer not to answer

**What do you consider to be your racial and/or ethnic background? (For example Zulu, Xhosa, Coloured, White, etc.)** _________________________

# SECTION 2: WOMEN'S REPRODUCTIVE HEALTH

## *2.1 Menstrual History*

**How old were you when you had your <u>first</u> menstrual period?** *Probe best estimate.*
Age in years: _________
- ❑ Don't know
- ❑ Prefer not to answer
- ❑ I never had a menstrual period

**When was the <u>last</u> day of your <u>last</u> menstrual period?** _______/________/______DAY/MONTH/YEAR

## *2.2 Vaginal Hygiene Practices*

**Which vaginal practices have you heard about? Please select all that apply.**
- ❑ Douching

- ❑ Cleansing with water
- ❑ Cleansing with soap
- ❑ Cleansing with detergents
- ❑ Cleansing with salt solution
- ❑ Cleansing with lemon juice
- ❑ Use of traditional herbs
- ❑ Insertion of creams
- ❑ Wiping with cloths/paper/etc.
- ❑ Other: _________

| | Have you ever… | | | If yes, how often have done this in the last month (weekly, daily, number of times)? | Why did you use the vaginal practice? Possible reasons see below. Please look at list below and name all numbers that apply. |
|---|---|---|---|---|---|
| | Yes | No | Prefer not to answer | | |
| Washed with water inside your vagina? | ❑ | ❑ | ❑ | | |
| Washed with soap inside your vagina? | ❑ | ❑ | ❑ | | |
| Washed with something else besides water or soap inside your vagina, including detergents or antiseptics? | ❑ | ❑ | ❑ | | |
| Used your fingers to wash inside your vagina? | ❑ | ❑ | ❑ | | |
| Used anything else to wash inside your vagina, including cloths or sponges? | ❑ | ❑ | ❑ | | |
| Put or kept traditional medicines or herbs inside your vagina? | ❑ | ❑ | ❑ | | |
| Put or kept medicine from a doctor/nurse/pharmacy inside your vagina? | ❑ | ❑ | ❑ | | |
| Put or kept paper, cloth, or cotton wool inside your vagina? | ❑ | ❑ | ❑ | | |
| Used a tampon? | ❑ | ❑ | ❑ | | |
| Put or kept anything else inside your vagina? | ❑ | ❑ | ❑ | | |
| Used anything to dry or tighten your vagina for sex? | ❑ | ❑ | ❑ | | |
| Practiced douching to clean your vagina? | ❑ | ❑ | ❑ | | |
| Had sex during your period? | ❑ | ❑ | ❑ | | |

**Why did you use these products?**
1) To clean the vagina
2) To reduce discharge
3) To reduce smell
4) To reduce itching
5) To clean blood after period
6) To avoid pregnancy
7) To reduce risk of sexually transmitted infections (e.g. Syphilis, gonorrhoea, herpes simplex virus type 2, chlamydia, etc.)
8) To reduce risk of HIV
9) For my own sexual pleasure
10) For my partner's sexual pleasure
11) Other: Please state reason

**Have you ever experienced any problems after using any of these products?**
- ❑ Yes
- ❑ No

- ❑ Don’t know
- ❑ Prefer not to answer

**If yes, what did you experience? Please select all that apply.**

- ❑ Itching or Irritation
- ❑ Pain
- ❑ Vaginal infections
- ❑ Bleeding
- ❑ Discomfort
- ❑ Other: ___________________________

**What do you think about using products in your vagina? Please select all that apply.**

- ❑ They are good for me
- ❑ They cause discharge or smell
- ❑ They increase my chances of getting an STI
- ❑ They increase my chances of getting or transmitting HIV
- ❑ My partner wants me to use them

## *2.3 Pregnancy History*

**Have you ever been pregnant? This includes all pregnancies, whether the outcome was a live birth, miscarriage, stillbirth, termination of pregnancy (abortion), or an ectopic/tubal pregnancy.** *Select one.*

- ❑ Yes
- ❑ No
- ❑ Prefer not to answer

**If yes**, how many times have you been pregnant? __________
**If yes**, when was the last pregnancy? (Month/Year) ______/_______

## *2.4 Contraceptive use*

**In the past have you used any form of contraception, safer sex method, or any other means to prevent pregnancy, to avoid getting or giving sexually transmitted infections, or to regulate your periods?**

- ❑ Yes
- ❑ No
- ❑ Don’t know
- ❑ Prefer not to answer

**In the past, what forms of contraception, safer sex method, or any other means to prevent pregnancy, to avoid getting or giving sexually transmitted infections, or to regulate your period have you used? Please select all that apply.**

- ❑ Oral contraceptive, also known as 'the pill'
- ❑ An injection/injectable, also known as 'Depo-Provera' or Nur-Isterate (circle correct one)
- ❑ NuvaRing, a vaginal ring that you insert once a month
- ❑ A contraceptive patch, also known as Ortho Evra and used once a week
- ❑ Implanon, also known as a "progestin implantable contraceptive"
- ❑ An Intrauterine Device, also known as an "IUD" or "Copper IUD”
- ❑ An Intrauterine System, also known as an "IUS" or "Mirena"
- ❑ A diaphragm, also known as a “cervical cap”
- ❑ The sponge
- ❑ Any vaginal creams, jellies, or foams
- ❑ Any emergency contraception, commonly known as "Escapelle", "the morning after pill", or "Norlevo"?

- ☐ Condoms
- ☐ The rhythm method, also known as “periodic abstinence”
- ☐ The withdrawal method
- ☐ Chosen to practice abstinence with male partners
- ☐ Any other method? (please specify) ______________________________________

**Do you smoke?**

- ☐ Yes
- ☐ No
- ☐ Don’t know
- ☐ Prefer not to answer

### *2.5 Vaginal discharge*

**Do you have vaginal discharge?**

- ☐ Yes
- ☐ No
- ☐ Don’t know
- ☐ Prefer not to answer

**If yes**, is it a chronic/long-term problem or a new/recent problem?

- ☐ Long-term
- ☐ New

**If yes**, what is the colour of your discharge? __________

**If yes**, what is the consistency of your discharge?

- ☐ Thick/cheesy
- ☐ Creamy
- ☐ Thin

**If yes**, what is the smell of your discharge?

- ☐ Strong
- ☐ Mild
- ☐ None

## SECTION 3: WOMEN'S SEXUAL HEALTH

The next section includes some personal questions about your sexual activities. Some of these questions are very personal and may make you feel uncomfortable. Since the survey is confidential, no one will know your answers. We would appreciate your participation in answering these questions as truthfully as possible. If you would like to complete this section yourself, you are welcome to do so. If you would like me to guide you through this whole section, that’s okay too.

**How old were you the first time you had vaginal sex?**

Indicate age in years: _________

- ☐ Don’t know
- ☐ Prefer not to answer

**How many different people have you had sex with in your lifetime?**

Number of different people: _________

- ☐ Don’t know

- ☐ Prefer not to answer

**During the past 6 months have you had vaginal sex?**
- ☐ Yes **If yes, with how many partners**? ____
- ☐ Don't Know
- ☐ Prefer not to answer

**During the past 6 months, have you had anal sex?**
- ☐ Yes **If yes, with how many partners**? ____
- ☐ No
- ☐ Don't Know
- ☐ Prefer not to answer

**During the past 6 months, have you had oral sex?**
- ☐ Yes **If yes, with how many partners**? ____
- ☐ No
- ☐ Don't Know
- ☐ Prefer not to answer

**How many of these relationships were "exclusive" or "mutually monogamous"** (meaning while you were in the relationship, you and your partner only had vaginal or anal sex with each other, and had no other sexual partners outside the relationship)? ______

**If you are using condoms with your partners, how often do you use them?**
- ☐ Always, every time I've had sex
- ☐ Most of the time
- ☐ Occasionally
- ☐ Never
- ☐ Unsure
- ☐ Prefer not to answer

**During the past 6 months, have you used condoms regularly?**
- ☐ Yes
- ☐ No
- ☐ Don't Know
- ☐ Prefer not to answer

**Did you use barrier protection (male or female condoms) with your last sexual act?**
- ☐ Yes
- ☐ No
- ☐ Don't Know
- ☐ Prefer not to answer

## *3.2 Risk factors*

**Have you ever been diagnosed with or been treated for a sexually transmitted infection?**
Examples: Syphilis, gonorrhoea, non-gonococcal urethritis, herpes simplex virus type 2 (HSV-2), chlamydia, pelvic inflammatory disease (PID), trichomonas, mucopurulent cervicitis, epididymitis, procitis, lymphogranuloma vernereum, chancroid, hepatitis B.
- ☐ Yes
- ☐ No
- ☐ Don't Know

- ❑ Prefer not to answer

**Have you or your partner had a STI in the past 6 months?**

- ❑ Yes
- ❑ No
- ❑ Don’t know
- ❑ Prefer not to answer

**How many sexually transmitted infections have you had in your lifetime?___________**

# Live biotherapeutics for treatment of bacterial vaginosis in HIV prevention in South African women – BV-trial1

**Principal Investigator:**
University of Cape Town, South Africa: Dr Shaun Barnabas

**Co-Investigators:**
University of Cape Town, South Africa: Dr Jo-Ann Passmore, Anna-Ursula Happel and Dr Heather Jaspan

**Funder:** Medicines Research Council

**University of Cape Town:**
Phone 021 650 7963

**Introduction**

Hello my name is ____________________. I am part of a research team from the University of Cape Town (UCT). You are taking part in a study comparing two different ways to treat bacterial vaginosis. When you have bacterial vaginosis, you have abnormal bacteria in your vagina. We would like to ask how you responded to the treatment. This information will be kept confidential and your personal information will not be shared with anyone.

# INTERVIEW DETAILS

**Participant Identification _____________**

**Date of Interview (DD/MMM/YYYY) _____________**

**Location of interview: _______________________**

**Interviewer Name: __________________________**

**Interview start time: (HH:MM AM/PM): ______________________**

**Interview stop time: (HH:MM AM/PM): ______________________**

## *1.1 Use of METRONIDAZOLE GEL*

**Have you used the Metronidazole gel?**

- ❑ Yes
- ❑ No
- ❑ Don't know
- ❑ Prefer not to answer

**If yes, did you complete the entire course?**

- ❑ Yes
- ❑ No
- ❑ Don't know
- ❑ Prefer not to answer

**If no, how many days did you take it for?** __________

**If no, why did you stop treatment?**

- ❑ Medication did not work
- ❑ Vaginal itching or irritation
- ❑ Vaginal pain
- ❑ Vaginal infections
- ❑ Vaginal bleeding
- ❑ Discomfort
- ❑ Other: ___________________________

**Have you ever experienced any problems after using the study product?**

- ❑ Yes
- ❑ No
- ❑ Don't know
- ❑ Prefer not to answer

**If yes, what did you experience? Please select all that apply.**

- ❑ Vaginal itching or irritation
- ❑ Vaginal pain
- ❑ Vaginal infections

- ❑ Vaginal bleeding
- ❑ Discomfort
- ❑ Other: ____________________________

## *1.2 Use of STUDY PRODUCT*

NOTE: PLEASE ONLY COMPLETE THIS SECTION IF YOU WERE GIVEN THE PROBIOTIC FOR VAGINAL HEALTH. IF YOU WERE ONLY GIVEN THE METRONIDAZOLE GEL, PLEASE GO TO SECTION 1.3

**Have you used the study product?**

- ❑ Yes
- ❑ No
- ❑ Don't know
- ❑ Prefer not to answer

**<u>If yes,</u> did you complete the entire course?**

- ❑ Yes
- ❑ No
- ❑ Don't know
- ❑ Prefer not to answer

**<u>If no</u>, how many days did you take it for? ___________**

**<u>If no,</u> why did you stop treatment?**

- ❑ Medication did not work
- ❑ Vaginal itching or irritation
- ❑ Vaginal pain
- ❑ Vaginal infections
- ❑ Vaginal bleeding
- ❑ Discomfort
- ❑ Other: ____________________________

**Have you ever experienced any problems after using the study product?**

- ❑ Yes
- ❑ No
- ❑ Don't know
- ❑ Prefer not to answer

**If yes, what did you experience? Please select all that apply.**

- ❑ Vaginal itching or irritation
- ❑ Vaginal pain
- ❑ Vaginal infections
- ❑ Vaginal bleeding
- ❑ Discomfort
- ❑ Other: ____________________________

**What do you think about using products in your vagina? Please select all that apply.**

- ❑ They are good for me
- ❑ They cause discharge or smell
- ❑ They increase my chances of getting an STI
- ❑ They increase my chances of getting or transmitting HIV
- ❑ My partner wants me to use them

## *1.3 Vaginal discharge*

**Has using the medication improved your discharge?**

- ❑ Yes
- ❑ No, there has been no change.
- ❑ No, symptoms worsened.
- ❑ Prefer not to answer

**<u>If yes,</u> how did the discharge improve?**

- ❑ Less discharge
- ❑ Change in colour
- ❑ Change is smell
- ❑ Don't know
- ❑ Other ___________

**<u>If symptoms have become worse</u>, what were the changes?**

- ❑ More discharge
- ❑ Change in colour
- ❑ Change is smell
- ❑ Don't know
- ❑ Prefer not to answer
- ❑ Other ___________

# Live biotherapeutics for treatment of bacterial vaginosis in HIV prevention in South African women – BV-trial1

**Principal Investigator:**
University of Cape Town, South Africa: Dr Shaun Barnabas

**Co-Investigators:**
University of Cape Town, South Africa: Dr Jo-Ann Passmore, Anna-Ursula Happel and Dr Heather Jaspan

**Funder:** Medicines Research Council

**University of Cape Town:**
Phone 021 650 7963

**Introduction**

Hello my name is _____________________. I am part of a research team from the University of Cape Town (UCT). You just finished taking part in a study comparing two different ways to treat bacterial vaginosis. When you have bacterial vaginosis, you have abnormal bacteria in your vagina. If you were in the group that was given the study product please complete this questionnaire.

In our study, you used the adjunctive probiotic for the treatment of bacterial vaginosis, and we would like to ask how you felt about using this treatment. This information will be kept confidential and will not be shared with anyone.

# INTERVIEW DETAILS

**Participant Identification ______________**

**Date of Interview (DD/MMM/YYYY) ____/____/______**

**Location of interview: ________________________**

**Interviewer Name: ___________________________**

**Interview start time: (HH:MM AM/PM): _______________________**

**Interview stop time: (HH:MM AM/PM): _______________________**

## *1. Use of the study product*

**During the study, did you like using the study product?**

- ❑ Yes
- ❑ No
- ❑ Don't know
- ❑ Prefer not to answer

**<u>If yes,</u> why did you like using it?**

________________________________________________________________________

**<u>If no</u>, why did you not like using it?**

________________________________________________________________________

**Would you use the study product again?**

- ❑ Yes
- ❑ No
- ❑ Don't know
- ❑ Prefer not to answer

**<u>If yes</u>, why would you use it again?**

- ❑ For prevention of vaginal discharge
- ❑ To treat vaginal discharge
- ❑ Prefer not to answer

**Would you recommend this product to other woman?**

- ❑ Yes
- ❑ No
- ❑ Don't know
- ❑ Prefer not to answer

**Did you use the product as recommended (oral, vaginal application)?**

- ❑ Yes
- ❑ No
- ❑ Don't know
- ❑ Prefer not to answer

**If no, why did you not use it as recommended?**

________________________________________________________________________

**Did you complete the entire course of the product?**

- ❑ Yes
- ❑ No
- ❑ Don't know
- ❑ Prefer not to answer

**If no, why did you not complete the course?**

________________________________________________________________________

## *2. Acceptability of the study product*

**How easy or difficult was the vaginal application of the product?**

- ❑ Easy
- ❑ Medium
- ❑ Difficult
- ❑ Don't know
- ❑ Prefer not to answer

**For the vaginal application of a product, which formulation would you prefer?**

- ❑ Spray
- ❑ Capsule
- ❑ Tablet
- ❑ Gel
- ❑ Tampon

**Did you prefer the oral or vaginal application?**

- ❑ Oral
- ❑ Vaginal
- ❑ Both applications
- ❑ Don't know
- ❑ Prefer not to answer

**Would you prefer a product with oral or vaginal application only?**

- ❑ Oral only
- ❑ Vaginal only
- ❑ Oral and vaginal
- ❑ Don't know

- ❑ Prefer not to answer

**How comfortable did you feel about others knowing that you use this product?**

- ❑ Very comfortable
- ❑ Comfortable
- ❑ Neither comfortable nor uncomfortable
- ❑ Uncomfortable
- ❑ Very uncomfortable
- ❑ No-one knew I was using the product
- ❑ Don't know
- ❑ Prefer not to answer

**Was it difficult to store the study product in your home?**

- ❑ Yes
- ❑ No
- ❑ Don't know
- ❑ Prefer not to answer

**Would you buy the product?**

- ❑ Yes
- ❑ No
- ❑ Don't know
- ❑ Prefer not to answer

**<u>If yes,</u> how much money would you be willing to spend?**

- ❑ Up to R25
- ❑ Up to R50
- ❑ Up to R100
- ❑ Up to R200
- ❑ Up to R300
- ❑ More than R300

**<u>If yes,</u> where would you want to buy the product?**

- ❑ Pharmacy
- ❑ Health store
- ❑ Grocery shop
- ❑ Clinic
- ❑ Other __________

**Who would you ask for advice regarding the use of probiotics?**

- ❑ Doctor
- ❑ Nurse
- ❑ Pharmacist
- ❑ Friends
- ❑ Partner
- ❑ Internet
- ❑ Other __________

**Do you believe that you received some benefit from using the product?**

- ❑ Yes
- ❑ No
- ❑ Don't know
- ❑ Prefer not to answer

**We are going to ask you some personal questions; you do not have to answer. You can choose to either answer these on your own or to have someone assist you to complete the questions.**

**Did you inform your partner(s) that you were using the product?**
- ❑ Yes
- ❑ No
- ❑ Prefer not to answer

**If yes, what was your partner(s) response to being informed that you were using the product?**
- ❑ Positive
- ❑ Negative
- ❑ Indifferent
- ❑ Prefer not to answer

**Did you have sexual intercourse while using the product?**
- ❑ Yes
- ❑ No
- ❑ Prefer not to answer

**If yes, did your partner notice during sexual intercourse that you were using the product?**
- ❑ Yes
- ❑ No
- ❑ Don't know
- ❑ Prefer not to answer

**Did the use of the product affect your sex life?**
- ❑ Yes
- ❑ No
- ❑ Prefer not to answer

**If yes, was the effect positive?**
- ❑ Yes
- ❑ No
- ❑ Prefer not to answer

**If yes, what was the positive effect?**

______________________________________________________________________

**If no, what was the negative effect?**

______________________________________________________________________
