# Supplementary figures and images for: Testing the regulatory framework in South Africa – a single-blind randomized pilot trial of commercial probiotic supplementation to standard therapy in women with bacterial vaginosis
Source: BMC Infect Dis. 2020 Jul 10;20:491. doi: 10.1186/s12879-020-05210-4 (PMC7350581; doi:10.1186/s12879-020-05210-4)

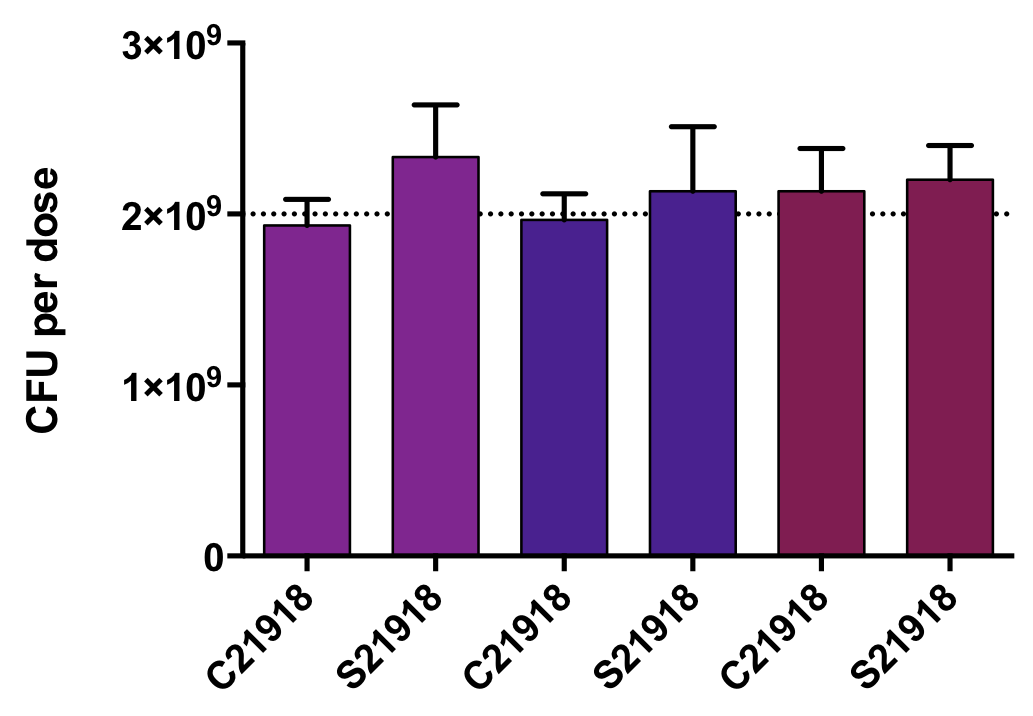

Supplement: Supplementary file 2 — Additional file 2: Figure S1. Bacterial concentration per dose unit of the commercial probiotic. The CFU per dose of three boxes (pink, purple and red) of the probiotic lot containing oral capsules (C) and vaginal spray (S) was determined using serial dilutions and compared to the manufacturers claim of 2 × 10^9^ CFU per dose (dotted line). [file 12879_2020_5210_MOESM2_ESM.tiff]
